# Nitrogen-Doped Porous Carbon Derived from Covalent Triazine Framework for Catalytic Oxidation of Benzyl Alcohol

**DOI:** 10.3390/nano14090744

**Published:** 2024-04-24

**Authors:** Xin Pan, Yanan Zhu, Yongchang Yang, Qianqian Zhu

**Affiliations:** 1 College of Chemistry and Chemical Engineering, Xi’an Shiyou University, Xi’an 710065, China; panxin070925@163.com (X.P.); yyc1073918900@163.com (Y.Y.); 2 College of Chemistry, Green Catalysis Center, Zhengzhou University, Zhengzhou 450001, China

**Keywords:** N-doped carbon, covalent triazine framework, NaCl, benzyl alcohol oxidation

## Abstract

The catalytic oxidation of alcohols is an important transformation in the chemical industry. Carbon materials with a large surface area and N doping show great promise as metal-free catalysts for the reaction. In this study, a rich N-containing covalent triazine framework polymerized by cyanuric chloride and p-phenylenediamine was used to synthesize N-doped porous carbon with the assistance of a pore-forming agent—NaCl. First, the mass ratio of the polymer/NaCl was optimized to 1:9. Then, the influence of the pyrolysis temperatures (700–1000 °C) on the materials was studied in detail. It was found that the carbon materials were gradually exfoliated by molten salt at high temperatures. XRD and Raman characterizations showed them with a certain graphitization. The optimal doped carbon CNN-1-9-900 achieved the highest surface area of 199.03 m^2^g^−1^ with the largest pore volume of 0.29 cm^3^g^−1^. Furthermore, it had a high N content of 9.9 at% with the highest relative proportion of pyridinic/graphitic N. Due to the synergistic effect between the surface area and pyridinic/graphitic N, CNN-1-9-900 showed the best performance for benzyl alcohol oxidation with TBHP at moderate conditions, and the process also worked for its derivatives.

## 1. Introduction

The catalytic oxidation of benzyl alcohol to benzaldehyde is one of the most fundamental reactions in organic synthesis and has wide applications in the fields of pharmaceuticals, agrochemicals, perfumes, dyes, and food additives [1,2,3]. Traditionally, benzyl alcohol was oxidized by stoichiometric permanganates, chromates, chromium trioxide or chromic acid [4]. For example, Lou et al. [5] used K_2_Cr_2_O_7_ as an oxidant to selectively convert primary alcohols to the corresponding aldehydes, while Jose et al. [6] selectively produced benzaldehyde from benzyl alcohol with KMnO_4_ as the oxidant. These systems caused serious pollution from the heavy metals. In contrast, H_2_O_2_, TBHP (tert-butyl hydroperoxide) and O_2_ are considered as green oxidants due to their environmental friendliness. Various heterogeneous catalysts based on noble metals (Pd, Au, Ru) or transition metals (Fe, Co, Zn, Ce, Cu, et al.) have been reported for this reaction with green oxidants [7]. Zhang et al. [8] synthesized a series of Pd/CeO_2_ catalysts by exposing different crystal facets of the CeO_2_ support and successfully activated O_2_ for benzyl alcohol selective oxidation by the most abundant oxygen vacancies and optimized metal-support interaction on the CeO_2_ (110) surface. Marelli et al. [9] prepared carbon-supported Au/CuO nanoparticles with core-shell heterostructures by the solvated metal atom dispersion method. A strong synergistic effect existed when the CuO shell only covered part of the Au surface at high Au/Cu molar ratios. Consequently, Au/CuO could effectively facilitate O_2_ activation for benzyl alcohol oxidation. Xu et al. [10] doped Sn into WO_3_/graphene to promote the interactions between the metal components and the graphene support, resulting in excellent performance for the selective oxidation of benzyl alcohol by H_2_O_2_. Iraqui et al. [11] synthesized spherical NiFe_2_O_4_ nanoparticles by co-precipitation followed by hydrothermal aging and found that nanoparticles with a size below 12 nm could work as efficient and reusable catalysts for benzyl alcohol conversion into benzaldehyde with TBHP as an oxidant under mild conditions. However, the high cost, easy aggregation and the detachment of the metals still limit their large-scale utilization. Under the circumstances, metal-free carbonaceous catalysts have received extensive attention in recent years as promising catalysts for selective oxidation of alcohols as well as other organic compounds owing to their low cost, excellent stability, diverse structures, and easy functionalization [12,13].

The catalytic abilities of carbon materials are closely related to their physical and chemical properties. First, the structure and morphology of carbon materials show great significance in their surface area and pore structure, which contribute to the exposure of active sites and the mass diffusion process [14,15]. Second, defects on the carbon surface greatly affect their electronic structure and thereby regulate catalytic performance [16,17,18]. Due to the differences in size and electronegativity, heteroatom doping is considered as a promising route to optimize the charge or spin distribution of carbon materials, thus promoting their catalytic performance [19,20]. Among various heteroatoms, the nitrogen atom is the most attractive dopant because it can be incorporated into multiple locations in the carbon framework without large lattice mismatch [21,22]. N-doped carbon was reported for benzyl alcohol oxidation by O_2_ [23], but it often required a high temperature (120 °C) and the yield was not satisfactory (23%, 5 h). In contrast, TBHP is a more efficient green oxidant. Lin et al. [13] found that N-doped nanodiamond could obtain a benzaldehyde yield of ~33.4% using TBHP under relatively mild conditions (70 °C, 4 h). However, the harsh preparation of nanodiamond limited its application. Therefore, it is still highly desirable to find a facile method to prepare N-doped carbon for benzyl alcohol catalytic oxidation by TBHP under moderate conditions.

Generally, N-doped carbon can be synthesized using two strategies, that is, post treatment and in situ doping [24,25]. Comparatively speaking, the in-situ approach dominated by pyrolyzing precursors with carbon and nitrogen atoms is more advantageous, due to the more uniform nitrogen distribution, higher yield rates and less time-consuming operations. Among the various precursors, N-containing polymers such as polydopamine, polyaniline and polyacrylonitrile are most often used to produce N-doped carbon [26,27,28]. Beyond these well-known ones, covalent triazine frameworks (CTFs) as novel nitrogen-rich polymers have recently attracted considerable attention. They are an important type of porous organic polymer built by 1, 3, 5-triazine rings with various guest functional groups, and have shown great potential in adsorption/separation [29], catalysis [30], energy storage and conversion [31], and drug delivery [32]. CTFs can be relatively easily synthesized by constructing triazine units or directly introducing triazine-containing monomers [33]. The unique structures endow the CTFs with high stability and porosity. More importantly, the presence of triazine nodes enriches the CTFs with N atoms, and incorporating N-containing guest groups can make the N content even higher [34,35,36]. Therefore, CTFs can be considered as a promising candidate for preparing N-doped carbon materials.

In this work, a covalent triazine framework formed from cyanuric chloride and p-phenylenediamine was directly calcinated with NaCl to synthesize N-doped porous carbon. The polymer acted as carbon and nitrogen sources, while NaCl acted as the porogen. After optimizing the mass ratio of the polymer/NaCl and the pyrolysis temperature, the resulting doped carbon achieved the highest surface area of 199.03 m^2^g^−1^ and the highest relative proportion of pyridinic/graphitic N doping. The optimal catalyst showed a benzaldehyde yield of 57.3% with TBHP as the oxidant at 80 °C/6 h, which was comparable to the previously reported N-doped carbon. This synthetic strategy is expected to provide a facile method to produce N-doped carbon for catalytic oxidation.

## 2. Materials and Methods

### 2.1. Materials

Cyanuric chloride (99%), p-phenylenediamine (99%), benzyl alcohol (99%), anisole (99.5%), tert-butyl hydroperoxide (70% in H_2_O) and 4-bromobenzyl alcohol (99%) were purchased from Aladdin (Shanghai, China). 4-Methylbenzyl alcohol (98%), 4-methoxybenzyl alcohol (98%), cinnamyl alcohol (98%), and 4-nitrobenzyl alcohol (98%) were purchased from Macklin (Shanghai, China). Methanol (anhydrous, 99.8%) was purchased from Sinopharm Chemical Reagent Beijing Co., Ltd. (Beijing, China). NaCl (≥99.5%) was purchased from Tianjin Damao Chemical Reagent Factory (Tianjin, China). All reagents were used as received without further purifications.

### 2.2. Catalyst Preparation

Synthesis of covalent triazine framework: first, 1.84 g of cyanuric chloride was dissolved in 50 mL of methanol. Then, under magnetic stirring at a speed of 500 rpm, another methanol solution of 30 mL which contained 1.62 g of p-phenylenediamine was added drop by drop. During the addition of p-phenylenediamine, white solids with a slight yellow color were gradually formed in the solution. After stirring for 4 h, the polymer was collected by vacuum filtration and dried under vacuum at 60 °C for 12 h. It was denoted as CNN.

Synthesis of N-doped carbon: 1 g of CNN was mixed with a certain amount of NaCl (3 g, 6 g, 9 g, and 12 g). After being ground thoroughly in an agate mortar, the mixture was placed in a tube furnace and heated at a rate of 3 °C/min to various temperatures under Ar and maintained at that temperature for 2 h. After cooling, the sample was dispersed in 100 mL of hot water by ultrasound and stirred for 2 h, and then collected by vacuum filtration. This operation was repeated 3 times to remove NaCl as much as possible and then dried at 60 °C overnight. For the compared sample without NaCl-assisted pyrolysis, 1 g of CNN was directly heated at a rate of 3 °C/min to the desired temperature and maintained under Ar for 2 h.

### 2.3. Catalyst Characterization

The morphologies and structures were studied using scanning electron microscope (SEM) and transmission electron microscopy (TEM). For SEM tests, the samples were fixed on the support and sputtered with Au-Pd using an Oxford Quorum SC7620 (Quorum, Lewes, UK) sputter coater under vacuum for 45 s, and then studied using a ZEISS Gemini 300 (Zeiss, Oberkochen, Germany) with a resolution of 0.6 nm @ 15 kV and 1.0 nm @ 1 kV. The acceleration voltage for imaging was 3 kV. EDS mappings of the materials were conducted on ZEISS Gemini 300 with a secondary electron detector (Oxford Xplore, Oxford Instruments, Oxford, UK) and the acceleration voltage was 15 kV. TEM and high-resolution TEM (HRTEM) images were obtained with a JEM-2100 (JEOL, Akishima-shi, Japan) with a point resolution of 0.23 nm and a lattice resolution of 0.14 nm, and the acceleration voltage was 200 kV. X-ray diffraction (XRD) was measured on a Bruker D8 Advance diffractometer with Cu Kα (λ = 0.154 nm) irradiation at 60 kV and 60 mA. The scanning range was 10–60° and the scanning rate was 10°/min. N_2_ adsorption–desorption isotherm tests were conducted on a gas adsorption analyzer (Micromeritics ASAP 2020, Micromeritics, Atlanta, GA, USA). The samples were first degassed under high vacuum at 200 °C for 12 h and then analyzed at liquid nitrogen temperature (77 K). The specific surface areas of the samples were calculated by Brunauer–Emmett–Teller (BET) method. XPS spectroscopy was carried out on Thermo Fisher Scientific K-Alpha (Waltham, MA, USA) spectrometer with an Al Kα X-ray irradiation (h*v* = 1486.6 eV) at 12 kV and 6 mA. The analysis chamber was equipped with self-calibration standards and the pressure in analysis was less than 2 × 10^−8^ mbar. A dual beam of ion and electron was used for charge neutralization. The binding energies were calibrated by shifting the C1s peak to 284.6 eV. Raman spectroscopy was measured on Thermo Scientific DXR instrument with a 532 nm laser.

### 2.4. Catalytic Activity Test

Substrates (0.5 mmol), catalyst (10 mg), TBHP (500 μL, 70% in water), and acetonitrile (2 mL) were added to a 15 mL pressure-resistant bottle sealed with a Teflon lid. The reaction was carried out at 80 °C for 6 h in an oil bath. After cooling, 50 μL anisole was added to the mixture as the internal standard. Then, the organics were extracted and analyzed using a gas chromatograph (GC7980, Techcomp, Hong Kong, China) with a flame ionization detector (FID) and a HB-5 capillary column (50 m in length and 0.32 mm in diameter). The identification was carried on GC-MS (Agilent 7890B/MSD-5977A, Agilent, Santa Clara, CA, USA) with an HP-5 capillary column (50 m in length and 0.2 mm in diameter).

## 3. Results and Discussion

**Scheme 1:**
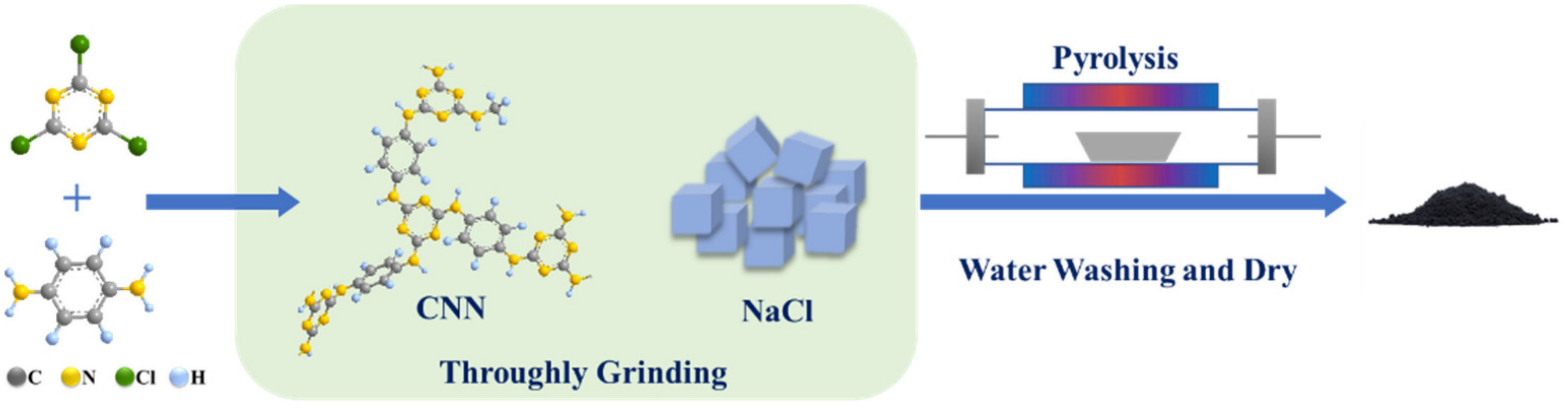
Illustration for preparing N-doped porous carbon via NaCl-assisted polymer pyrolysis.

The N-doped porous carbon was synthesized via a NaCl-assisted CTF pyrolysis method as shown in Scheme 1. First, the precursor covalent triazine framework was synthesized by the polymerization of cyanuric chloride and p-phenylenediamine and denoted as CNN. Due to the unsaturated C-N backbone, the C-Cl bonds in cyanuric chloride exhibit high reactivity and are easily attacked by nucleophiles. When p-phenylenediamine was added to the methanol solution of cyanuric chloride, a nucleophilic substitution reaction occurred between them. Because amine groups were present both in the head and tail of p-phenylenediamine, a cross-linked network structure with abundant nitrogen atoms finally formed [37]. Subsequently, the polymer CNN (1 g) was ground thoroughly with NaCl in mass ratios of 1:3, 1:6, 1:9 and 1:12. Then, the mixtures were pyrolyzed at a certain temperature under Ar. During the process, CNN performed as the carbon and nitrogen sources, while NaCl was used as a template for pore-formation. After calcination and washing fully to remove NaCl, the N-doped porous carbon materials were obtained and denoted as CNN-1-X-Y, X representing the mass of NaCl (g), Y representing the calcination temperature (°C).

**Figure 1:**
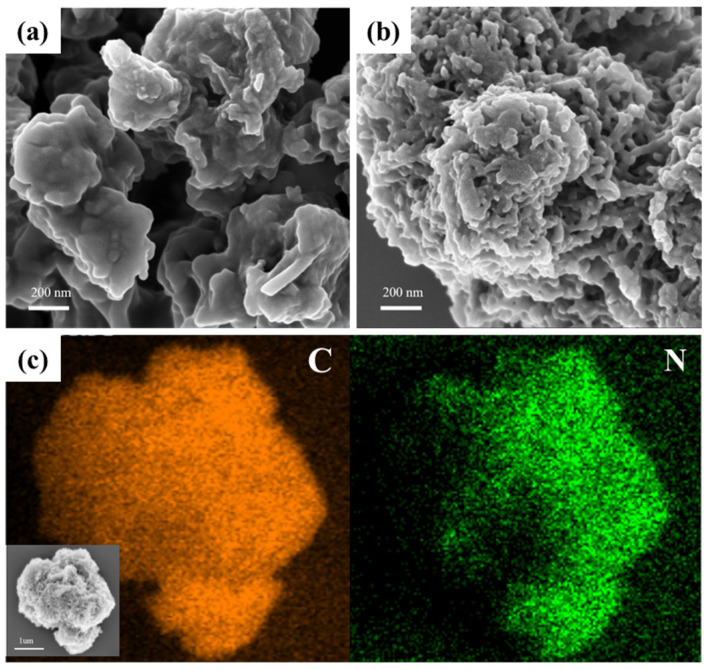
SEM images of (**a**) CNN-800, (**b**) CNN-1-9-800, (**c**) EDS mapping image of CNN-1-9-800.

**Figure A1:**
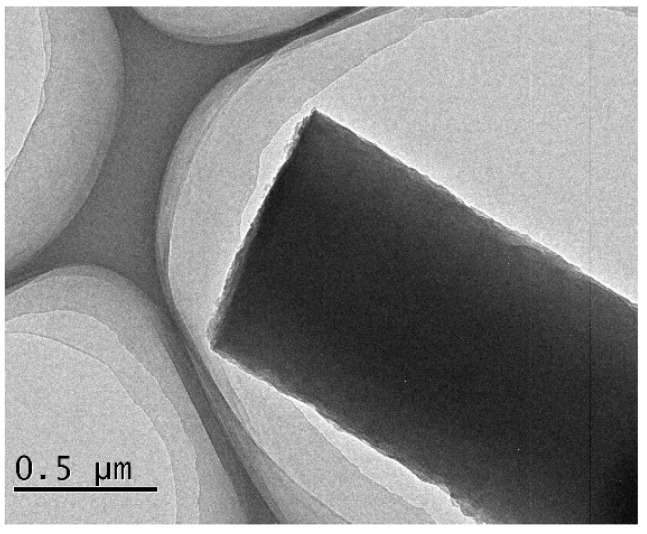
TEM image of CNN-800.

**Figure A2:**
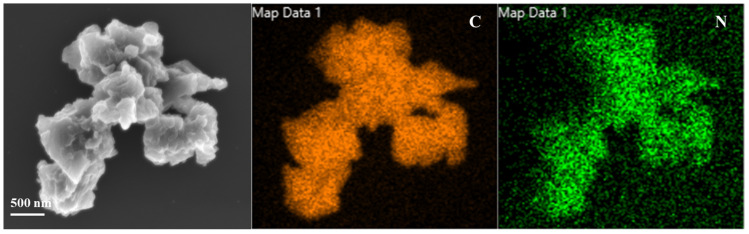
EDS mapping image of CNN-800.

First, samples calcinated at 800 °C were taken as examples to study the pore-forming effect of NaCl. The SEM image (Figure 1a) showed that without the assistance of NaCl, the polymer after pyrolysis (denoted as CNN-800) appeared as an irregular and dense structure. The TEM image with low electron transmittance (Figure A1) also showed it had a thick block structure, attributed to the melting and shrinking of the polymer chain under high temperature. Interestingly, when NaCl was introduced, it started to partially melt at 800 °C (melting point of NaCl: ~801 °C) and acted in situ as a structure-direct agent to inhibit the shrinkage of the polymer chain. In fact, previous studies have shown that molten NaCl can work as a reactive chloride salt to react with carbon under high temperatures for the creation of pores, during which Cl^−^ ions as etching agents also engage in the pore-forming process [38,39]. Therefore, the sample (CNN-1-9-800 as an instance, Figure 1b) turned out to be a cluster structure with intersecting branches and obvious holes, demonstrating the necessity of using NaCl as pore-forming agent. Energy dispersive spectroscopy (EDS) mapping images (Figure 1c and Figure A2) indicated that N atoms were evenly distributed both on CNN-800 and CNN-1-9-800, proving their successful doping, but the N content was increased from 14.97% in CNN-800 to 15.76% in CNN-1-9-800 which was consistent with the previous report that molten salt was able to avoid the loss of nitrogen [40].

**Figure 2:**
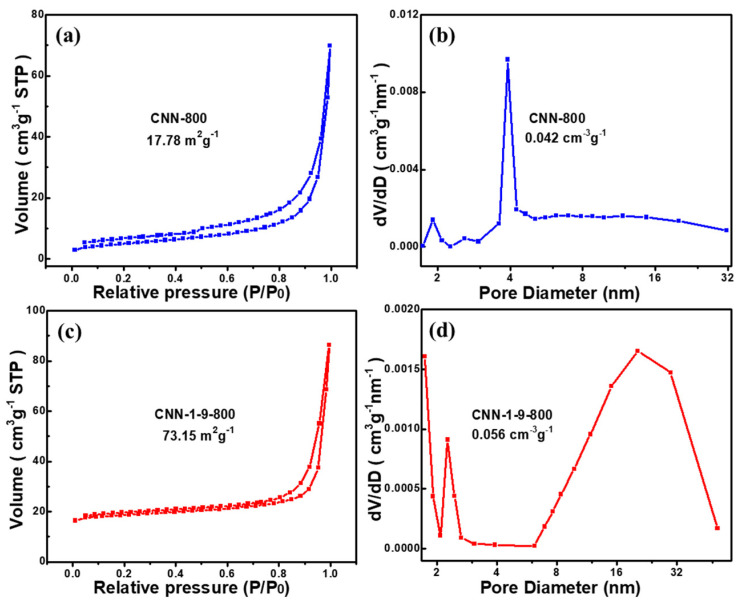
N_2_ adsorption–desorption isotherms and the corresponding BJH pore size distributions of (**a**,**b**) CNN-800, (**c**,**d**) CNN-1-9-800.

N_2_ adsorption–desorption isotherms were further characterized to verify the important role of NaCl. It can be seen from Figure 2a, b that when CNN was pyrolyzed alone at 800 °C, the resulting carbon material had a specific surface area as low as 17.78 m^2^g^−1^ with a pore volume of only 0.042 cm^3^g^−1^. The corresponding BJH pore size distributions showed the pores were mainly centered at 3.9 nm. However, an intensive peak at this location was commonly caused by the tensile strength effect in N_2_ adsorption. It was a false peak which should be ignored. After calcinating with NaCl, the surface area of CNN-1-9-800 was promoted more than fourfold to 73.15 m^2^g^−1^ and its pore volume rose to 0.056 cm^3^g^−1^, accompanied by large variations in pore diameters (Figure 2c,d), indicating the significant pore-making effect of NaCl.

**Figure 3:**
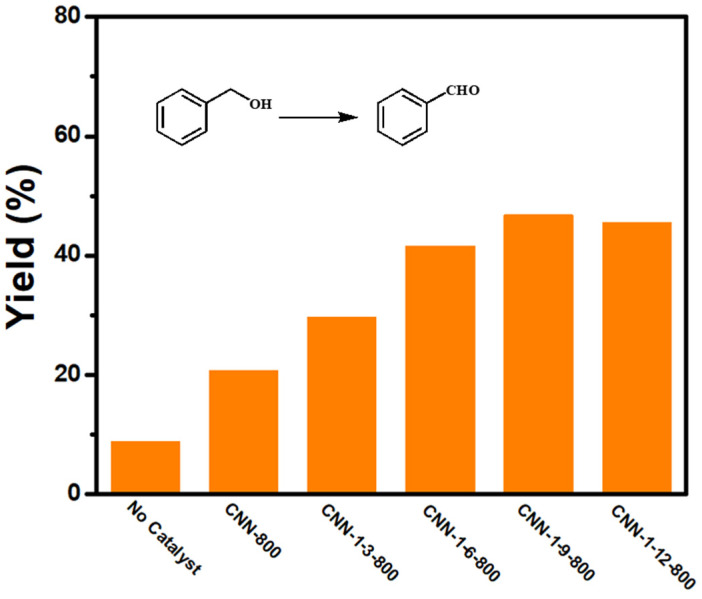
Catalytic performances of different carbon catalysts for benzyl alcohol oxidation. Reaction conditions: catalyst (10 mg), substrate (0.5 mmol), TBHP (500 uL), solvent (2 mL), 80 °C, 6 h.

The benzyl alcohol selective oxidation with TBHP was performed to evaluate preliminarily the advantage of using salt in the catalyst preparation. As shown in Figure 3, there was only an 8.9% yield of benzaldehyde obtained without the catalyst. When CNN-800 was applied to this system, it worked out for this reaction, but the yield was only increased to 20.8%, due to the unsatisfactory specific surface area and pore volume. With the introduction of NaCl to create pores in the preparation process, the catalytic performances were gradually improved and attained the highest yield of 46.8% when the mass ratio of CNN/NaCl was 1:9, demonstrating NaCl-assisted pyrolysis plays an indispensable role in improving the performance of N-doped carbon.

**Figure 4:**
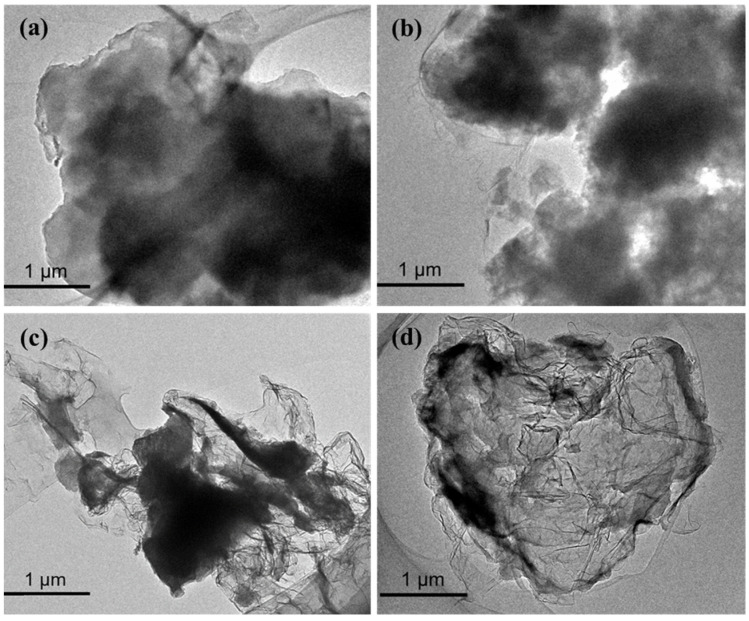
TEM images of (**a**) CNN-1-9-700, (**b**) CNN-1-9-800, (**c**) CNN-1-9-900, (**d**) CNN-1-9-1000.

**Figure A3:**
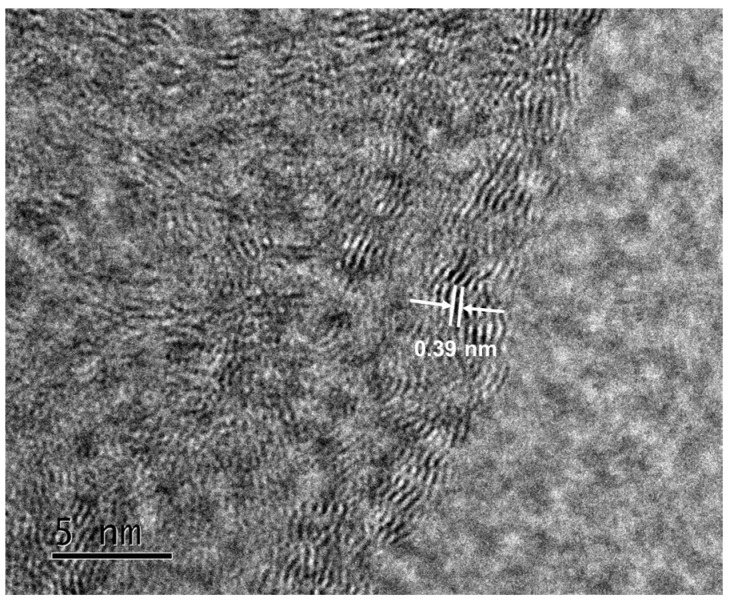
HRTEM image of CNN-1-9-900.

Based on the optimum mass ratio of CNN/NaCl, the effect of the pyrolysis temperature (700–1000 °C) on the carbon materials was further studied. First, TEM images showed that the temperatures exerted a great influence on the final morphologies of CNN-1-9-Y. The carbon material that was produced at 700 °C presented a blocky structure (Figure 4a). As discussed above, when the temperature was increased to 800 °C, NaCl started to melt and acted as an in-built template to protect the adjacent carbon from fusion and agglomeration. The resulting material CNN-1-9-800 (Figure 4b) obviously became loose and some small nanosheets began to appear around the edges. Further increasing the temperature to 900 °C, the liquid molten salt could be better impregnated into the carbonizing polymers and work as a soft template in situ to promote the aggregated carbon from exfoliating into lamellar layers [40,41]. The TEM image (Figure 4c) demonstrates that the carbon derived at this temperature was no longer a single block structure, but partially presented as nanosheets. The HRTEM image (Figure A3) displays clear graphitic layers at the edge of the nanosheets with a spacing of 0.39 nm, larger than the common value (0.34 nm) of the (002) plane of graphite [42,43]. When the temperature was finally raised to 1000 °C, the penetration of the molten NaCl into the agglomerative carbon was enhanced, leading to an almost complete exfoliation of the carbon into nanosheets (Figure 4d).

**Figure 5:**
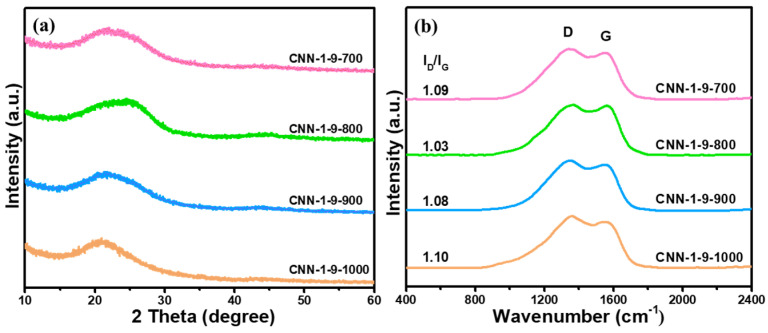
(**a**) XRD patterns and (**b**) Raman spectra of CNN-1-9-Y.

The XRD patterns of CNN-1-9-Y (Figure 5a) showed that all of them had a weak and broad peak at 2θ = 20.8–25.5°, implying that they were mainly composed of amorphous carbon but with a certain degree of graphitization [44,45]. As the temperature increased, the diffraction peaks shifted first to the right and then to the left, meaning an opposite change in the interlayer spacings which were closely related to the defects [46]. Moreover, since the 2θ angles of these diffraction peaks were all lower than 26.4° which corresponded to the (002) plane of graphite, the interlayer spacings of graphitic carbon in all samples were larger than 0.34 nm. The Raman spectra (Figure 5b) were consistent with the XRD results. The band at 1345 cm^−1^ corresponded to the disordered carbon (D band), whereas the band at 1580 cm^−1^ represented the graphitic carbon (G band) [47,48]. The intensity ratio I_D_/I_G_ was 1.09, 1.03, 1.08 and 1.10, respectively, for CNN-1-9-Y (700–1000 °C). It suggested that defects in the carbon decreased first and then increased gradually with the temperature, which was probably because more carbon became structurally ordered from 700 to 800 °C as the salt changed from solid to molten, but when continued to a higher temperature, more N atoms began to enter the carbon layer that induced more defects and larger spacing [23,49]. It was consistent with the previous report that molten salt was beneficial for the formation of defects and facilitated N incorporating into the carbon [40].

**Figure 6:**
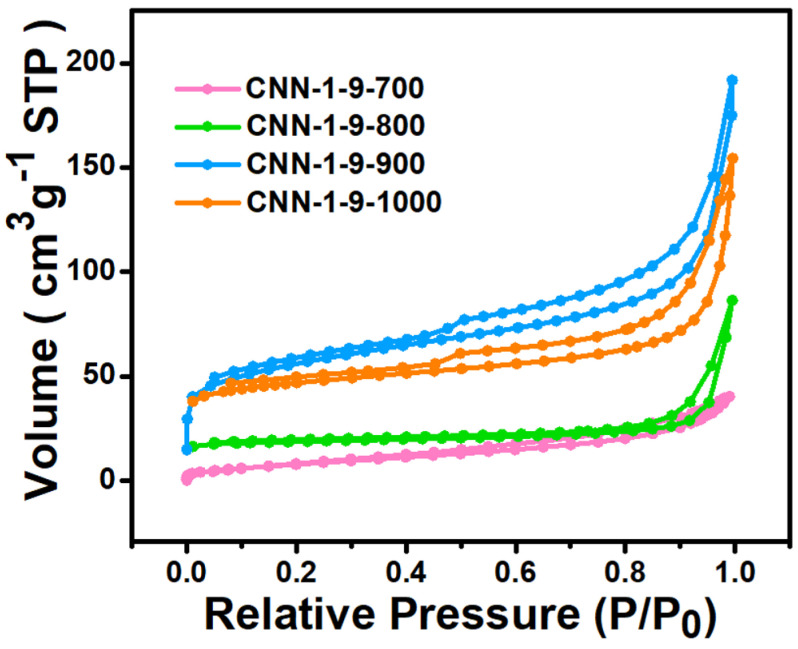
N_2_ adsorption–desorption isotherms of CNN-1-9-Y.

N_2_ adsorption–desorption tests of CNN-1-9-Y were conducted to investigate the carbonization temperature effects. The corresponding isotherms (Figure 6) indicated that the surface areas of the N-doped carbon could be facilely regulated by changing the temperatures. All the carbon materials exhibited an isotherm with typical type-IV characteristics. Among them, the samples CNN-1-9-700 and CNN-1-9-800 displayed an H3-type hysteresis loop, associated with mesopores inherent in the carbon structures as well as those from their accumulation [50]. In contrast, CNN-1-9-900 and CNN-1-9-1000 presented H4-type hysteresis, indicating the presence of micropores and mesopores [51]. When the temperature was gradually increased from 700 °C to 900 °C, the BET specific surface area (Table A1) was correspondingly promoted from 30.34 m^2^g^−1^ for CNN-1-9-700 to 199.03 m^2^g^−1^ for CNN-1-9-900, and the pore volume was raised from 0.045 cm^3^g^−1^ to 0.29 cm^3^g^−1^, contributing to the gradual exfoliation of the carbon materials and leading to an increase in the number of active sites. However, continuing to raise the temperature, the surface area of CNN-1-9-1000 decreased to 148.33 m^2^g^−1^ with a pore volume of 0.24 cm^3^g^−1^, probably because some of the pores collapsed at such a high temperature [12].

**Figure 7:**
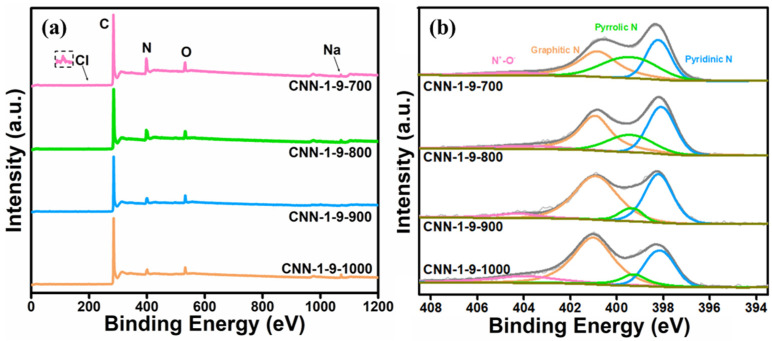
(**a**) XPS survey spectra and (**b**) high-resolution N 1s spectra of CNN-1-9-Y.

**Figure A4:**
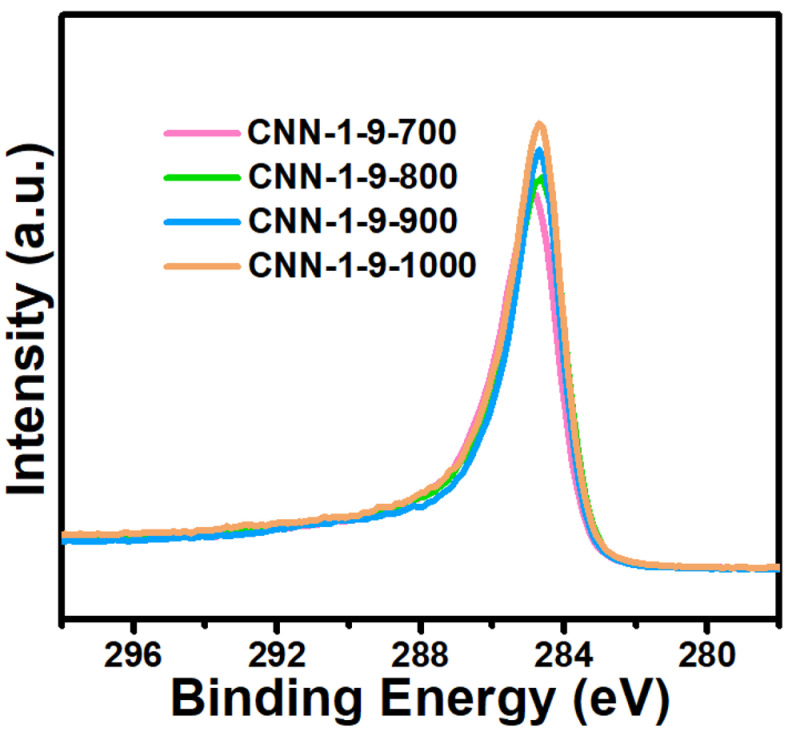
High-resolution C 1s spectra of CNN-1-9-Y.

**Figure A5:**
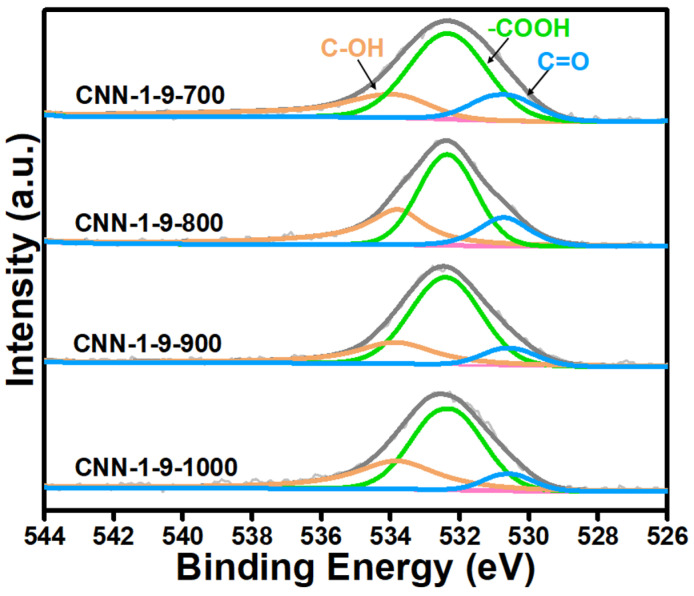
High-resolution O 1s spectra of CNN-1-9-Y.

Further, XPS analyses were performed to study the chemical compositions and chemical states of the N-doped carbon produced at different temperatures. The XPS survey spectra (Figure 7a) substantiated the co-existence of C and N atoms in the materials. The samples obtained at 700–1000 °C all showed typical sp^2^ C 1s spectra (Figure A4), demonstrating their certain graphitization [52]. The content of N atoms on the carbon surface (Table A1) were 16.1 at%, 13.7 at%, 9.9 at%, and 7.1 at%, respectively, for CNN-1-9-700, CNN-1-9-800, CNN-1-9-900, and CNN-1-9-1000. Obviously, N atoms were more likely to be lost at high pyrolysis temperatures, but due to the abundant N atoms in the polymer precursor and the retention effect of the molten salt, the N contents were still higher than most materials reported to be synthesized at the same temperature [53,54,55]. Notably, O atoms were also observed in the survey spectra. Because the precursor itself contained no oxygen, these O atoms were considered to come from the inevitable trace oxidation and adsorption of water vapor when the materials contacted with air [56]. The high-resolution O 1s spectra (Figure A5) could be deconvoluted into three peaks, demonstrating that the O atoms existed in the form of C=O group (530.7 eV), -COOH group (532.4 eV), and C-OH group (533.9 eV) [57,58]. In addition, trace amounts of Na and Cl atoms were also present in the survey spectra. Since NaCl had been removed with hot water as much as possible, these residues should be caused by trace Na^+^ and Cl^−^ ions (mainly Na^+^ ions) embedded in the graphitized carbon layer at high temperatures, which may explain why the interlayer spacings of the graphitic carbon in CNN-1-9-Y were larger than 0.34 nm. In fact, the popular sodium-ion battery studies have confirmed that Na^+^ intercalation between carbon layers can indeed expand the layer spacing of graphite [59,60]. For the selective oxidation of benzyl alcohol by N-doped carbon, most studies reported that the active species were pyridinic or graphitic N [13,14]. Therefore, the states of N atoms in CNN-1-9-Y were investigated in detail by N 1s spectra (Figure 7b). All the spectra could be divided into four peaks, attributed to pyridinic N (398.3 eV), pyrrolic N (399.5 eV), graphitic N (401.0 eV), and oxidized N (404.1 eV), respectively [12,13]. The sums of the relative proportions of pyridinic N and graphitic N in the total N atoms (Table A2) were 69.8%, 72.7%, 85.0%, and 82.1%, respectively, for CNN-1-9-700, CNN-1-9-800, CNN-1-9-900, and CNN-1-9-1000.

**Figure 8:**
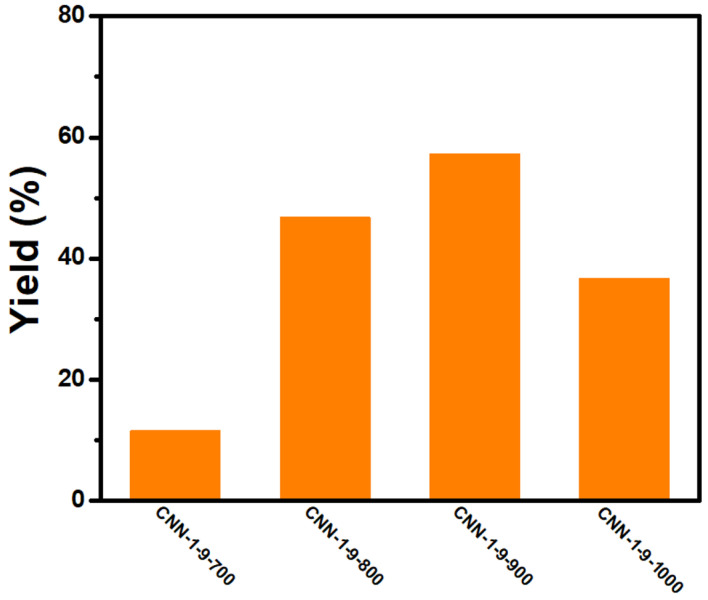
Catalytic performance of CNN-1-9-Y, reaction conditions: catalyst (10 mg), substrate (0.5 mmol), TBHP (500 uL), solvent (2 mL), 80 °C, 6 h.

Afterwards, the catalytic oxidation of benzyl alcohol with TBHP under 80 °C was conducted again to study the influence of pyrolysis temperature on the performance of CNN-1-9-Y. It can be seen from Figure 8 that there was a volcanic relationship between the yield and the pyrolysis temperature, and the most suitable temperature for preparing the catalyst was 900 °C. The benzaldehyde yield at 6 h increased gradually from 11.6% for CNN-1-9-700 to 57.3% for CNN-1-9-900, and then decreased to 36.8% for CNN-1-9-1000. Based on the above characterizations, the optimal performance of CNN-1-9-900 can be ascribed to the synergistic effect between the highest surface area and the relatively high proportion of pyridinic/graphitic N, in which the surface area contributes to the exposure of more active sites and faster mass transportation, while pyridinic/graphitic N atoms contribute to regulating the electronic structure of carbon. Finally, the catalytic oxidation of different primary alcohols over CNN-1-9-900 were explored (Table 1). This analysis showed that the catalyst was active for different p-substituent benzyl alcohols. When Br-, CH_3_O-, -CH_3_, -NO_2_ existed on the para-site of benzene ring, the yield of the corresponding aldehydes at 80 °C/6 h was 66.2%, 38.7%, 30.0%, and 41.9%, respectively. This was also the case for the oxidation of unsaturated alcohol. The yield of cinnamyl alcohol to cinnamaldehyde was 37.1%.

## 4. Conclusions

In summary, N-doped porous carbon catalysts were synthesized by pyrolyzing a kind of nitrogen-rich covalent triazine framework with the assistance of molten NaCl. The polymer acted as the carbon and nitrogen sources, while NaCl worked as the porogen. The pore-making effect of NaCl was confirmed and the optimal mass ratio of polymer/NaCl was identified as 1:9. Under this mass ratio, the effect of the pyrolysis temperatures on the N-doped carbon was studied. It was found that increasing the temperature could facilitate molten salt to peel the bulk carbon into nanosheets. XRD and Raman characterizations showed them with a certain graphitization. The best pyrolysis temperature was 900 °C. The resulting CNN-1-9-900 had the largest surface area of 199.03 m^2^g^−1^ with the largest pore volume of 0.29 cm^3^g^−1^. Furthermore, it had a high N content of 9.9 at% and the highest relative proportion of pyridinic/graphitic N of 85.0% obtained from XPS. The synergistic effect between the surface area and pyridinic/graphitic N gave CNN-1-9-900 the optimal catalytic performance, which could obtain a benzaldehyde yield of 57.3% using TBHP at moderate conditions (80 °C/6 h). This method also worked for the benzyl alcohol derivatives. The synthesis strategy is expected to provide a facile way to prepare N-doped carbon materials as metal-free catalysts for catalytic oxidation.

## Figures and Tables

**Table 1:** Catalytic oxidation of different alcohols over CNN-1-9-900.

Entry	Substrate	Product	Time (h)	Yield (%)
1	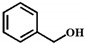	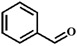	6	57.3
2	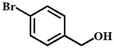	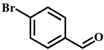	6	66.2
3	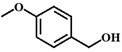	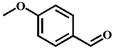	6	38.7
4	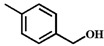	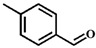	6	30.0
5	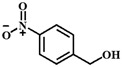	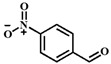	6	41.9
6	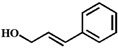	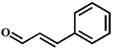	6	37.1

Reaction conditions: catalyst (10 mg), substrate (0.5 mmol), TBHP (50 uL), solvent (2 mL), 80 °C, 6 h.

## Data Availability

Data are contained within the article.

## Appendix A

**Table A1:** Surface area and pore volume of CNN-1-9-Y.

Sample	*S*_BET_ (m^2^g^−1^)	*V*_t_ (cm^3^g^−1^)
CNN-1-9-700	30.34	0.045
CNN-1-9-800	73.15	0.056
CNN-1-9-900	199.03	0.29
CNN-1-9-1000	148.33	0.24

**Table A2:** Total N contents (at%) and relative proportion (%) of different N species in CNN-1-9-Y.

Sample	N (Total, at%)	Relative Proportion of Different N Species in the Total N				
		Pyridinic N + Graphitic N (%)	Pyridinic N (%)	Graphitic N (%)	Pyrrolic N (%)	N^+^-O^−^ (%)
CNN-1-9-700	16.1	69.8	39.7	30.1	22.2	8.0
CNN-1-9-800	13.7	72.7	34.2	38.5	20.8	6.5
CNN-1-9-900	9.9	85.0	32.3	52.7	8.4	6.6
CNN-1-9-1000	7.1	82.1	27.2	54.9	8.1	9.8
